# What’s inside the Mind of a CEO? The Effects of Discretionary Slack Resources on R&D Investment

**DOI:** 10.3390/bs13030247

**Published:** 2023-03-11

**Authors:** Seunghye Lee, Won-Yong Oh, Young Kyun Chang

**Affiliations:** 1 Sogang Business School, Sogang University, Seoul 04107, Republic of Korea; 2 Lee Business School, University of Nevada, Las Vegas, NV 89154, USA

**Keywords:** CEO, R&D investment, slack resources, promotion focus, future focus, aspiration level

## Abstract

This study investigates the effects of discretionary slack resources on a firm’s R&D investment. Specifically, we examine whether and how a CEO’s psychological traits play a role in the relationship between discretionary slack and R&D investment. Using a panel sample from U.S. manufacturing firms in 2006–2010, we found that slack resources lead to an increase in R&D intensity. Furthermore, this positive effect is stronger when CEOs have a strong promotion focus and perceive an addressable negative attainment discrepancy. Thus, our findings show how (a) the level of discretionary slack and (b) CEOs’ promotion focus and aspiration level jointly shape R&D investment decisions. Considering CEOs’ psychological traits contributes to a more comprehensive view of the conditions under which discretionary slack resources matter for a firm’s strategic decisions.

## 1. Introduction

There has been extensive research on the impact of slack resources on a firm’s innovation-seeking behaviors (e.g., [1,2,3]). Most previous studies examining the linkage between slack and innovation are based on the behavioral theory of the firm (e.g., [4]), arguing that organizational slack plays a stabilizing and an adaptive role [5]. However, relatively little research has paid attention to the behavioral aspects of the key decision maker, while the key assumption of the behavioral theory of the firm is that a firm’s strategic behavior is represented by a manager’s decision. As such, in this paper, we focus on the roles of CEOs’ psychological traits in the relationship between discretionary slack and a firm’s research and development (R&D) investment, because organizational slack alone may not fully explain the variance in a firm’s spending on R&D investment.

The decision to invest in R&D activities as input for innovation has been considered one of a CEO’s most important decisions [6]. Since the CEO is the person responsible for the decision making of the entire organization [7], a number of studies (e.g., [8,9]) have focused on how CEO characteristics affect a firm’s strategic choices. Specifically, recent studies have focused increasingly on the roles of CEOs’ psychological traits and cognition (e.g., [10,11,12,13]). We build our arguments from and add to this body of research, based on the upper echelons perspective, by examining the roles of CEOs’ psychological traits and cognition in a firm’s strategic decisions.

In this study, we assume that decisions on how to allocate slack resources for R&D investment can be influenced by the CEO’s psychological traits (e.g., [14]), which are related to the key characteristics of R&D investment: (1) promotion focus, (2) future focus, and (3) aspiration level. Given that R&D investment has outcome uncertainty and a high risk of failure [15], a CEO’s level of promotion focus (i.e., obtaining positive results from actions) matters. Also, R&D investment requires a willingness to invest with a long-term perspective [2], and thus CEOs should be attentive to future outcomes over the long term, as reflected by their future focus. Lastly, R&D investment is an organizational search behavior as a response to performance feedback [1,16], and thus CEOs are attentive to the aspiration level, as they are eager to close the gap between performance deficiencies compared to meaningful reference points (i.e., attainment discrepancy).

In this regard, we predict that the relationship between a firm’s discretionary slack and R&D investment would be patterned differently depending on these psychological traits of CEOs. Using panel data from publicly traded U.S. manufacturing firms, we found that discretionary slack is positively related to a firm’s R&D investment and this pattern is more pronounced when CEOs have a high level of promotion focus to gain positive outcomes. Furthermore, we found that the relationship is stronger when CEOs face an achievable gap in their aspiration levels (i.e., a small negative attainment discrepancy), which is consistent with the attention-based view [17], suggesting that managers tend to switch the focus of their attention from aspiration to survival in financial distress.

Therefore, our findings suggest that CEOs’ psychological traits play a critical role in a firm’s decision making related to allocating discretionary slack resources. Based on these findings, this study makes several important contributions. First, it provides evidence of how the microfoundation perspective [18] complements the behavioral theory of the firm [5]. Our findings suggest the importance of the key decision maker’s cognition in shaping firm-level decisions. As such, this study suggests that individual-level psychological factors play important roles in predicting organizational decisions. Second, this study makes unique contributions to the field of management studies on R&D investment by examining the moderating role of CEOs’ psychological traits. Particularly, our results show that the positive effects of discretionary slack on a firm’s R&D investment are strengthened when CEOs have a high focus on promotion. Furthermore, we found that the positive relationship between discretionary slack and R&D investment is stronger when the firm performs below the aspiration level, but the gap is moderate. This is consistent with the attention-based view [17], which suggests that decision makers shift their focus of attention depending on the degree of attainment discrepancy (e.g., [19]). Based on these findings, this study sheds additional light on our understanding of a firm’s investment decisions, drawing on the behavioral theory of the firm, the upper echelons perspective, and the attention-based view.

## 2. Theoretical Background

### 2.1. Discretionary Slack and R&D Investment

Slack resources are defined as “the disparity between the resources available to the organization and the payments required to maintain the coalition” [5] (p. 36). Organizational slack can not only cushion the firm from internal or external pressures [20], but it can also allow firms to enact a strategy with respect to environmental changes [21]. While slack resources play a key role in a firm’s strategic choices, in this study, we specifically focus on the discretionary slack [20,22], which could directly affect a firm’s strategic choices. Unlike the organizational slack with low discretion, such as fixed assets and debt [21], discretionary slack, such as cash, represents resources which the manager has more discretion to redeploy.

There have been numerous studies (e.g., [2,23,24,25,26,27]) examining the relationship between a firm’s slack resources and its innovation-seeking investments. In particular, discretionary slack, such as cash, has increasingly become an important factor when managers make R&D decisions [25,27]. It has been assumed that organizational slack serves as a “buffer” from financial shortages [5]. As such, slack provides opportunities to conduct experimental projects with uncertainty [20], which gives rise to innovational activities, such as developing new products [23], engaging in corporate entrepreneurship [28], and undertaking acquisitions [29]. In other words, organizational slack allows managers to take risks by initiating something new for their firms’ performance improvement.

However, previous studies (e.g., [3,30]) suggest that the relationship between organizational slack and innovation-seeking behaviors is not uniformly positive or negative. This is because organizational slack serves as a source of funding for innovation (i.e., slack search) or insufficient slack triggers search for a solution (i.e., problemistic search). Thus, a number of studies (e.g., [3,31]) suggest the curvilinear relationship between organizational slack and R&D investment, indicating that too little or too much slack may discourage innovation-seeking behaviors. Taken together, the existing literature shows inconclusive empirical evidence regarding how organizational slack affects innovation-seeking behaviors. The inconclusive evidence implies that organizational slack alone does not fully explain the variance in a firm’s spending on R&D. Thus, this paper joins the debate by taking top executives’ behavioral tendencies or cognitive mindsets into account. We pay particular attention to the psychological traits of CEOs regarding R&D investment decisions.

### 2.2. CEO’s Psychological Traits

An individual’s psychological traits lay a pathway for an individual’s motivations [32], goal selection [33], and risk taking [34]. As such, we argue that decision making on how to allocate discretionary slack resources for R&D investment will be affected by the CEOs’ psychological traits: (1) promotion focus, (2) future focus, and (3) aspiration level. We focus on these psychological traits because they are closely related to a firm’s R&D decisions (e.g., [14]) due to the unique attributes associated with R&D investment. R&D investment requires that a decision maker has a gain-seeking tendency, a long-term time horizon, and the ability to adapt to negative attainment discrepancy (i.e., performance below his or her aspiration).

#### 2.2.1. Regulatory Focus

R&D investment has a high level of outcome uncertainty and risk of failure (e.g., [15]). For example, the risk of failure in terms of technical (e.g., technology obsolescence), competitive (e.g., competitor’s acquisition of patent being used in a project), and market (e.g., failure in commercialization to meet consumer demand) aspects is inherently associated with R&D investment [35]. Thus, the CEO’s attitude of paying attention to the potential gain vs. loss matters in the relationship between discretionary slack and R&D investment. Regulatory focus [32] describes two distinctive self-regulatory modes (i.e., promotion focus and prevention focus) associated with how individuals tend to seek pleasure and avoid pain. In general, a promotion focus is concerned with obtaining positive results through achievement, growth, and gain, whereas a prevention focus is concerned with avoiding negative outcomes, specifically related to safety, stability, and protection [36].

Individuals with a promotion focus pursue growth and advancement, whereas individuals with a prevention focus seek security and safety [33,37]. Such motivational differences also affect an individual’s attitude toward achieving their goals. Promotion-focused people tend to pursue their goals as their “ideal” selves, which reflect their hopes, aspirations, and wishes [38], whereas prevention-focused people try to attain their goals with their “ought” selves, and focus more on duties and responsibilities [12]. Such differences also influence an individual’s risky decision-making strategy (e.g., [12,14,39,40]). For example, Higgins and Spiegel [41] found that promotion-focused people are more concerned with opportunities for gains, and thus prefer risk-taking actions (e.g., realizing financial benefits from R&D investment). In contrast, people with a strong prevention focus are concerned with avoidance of losses, and thus are more risk-averse (e.g., minimizing financial loss in a case of R&D failure). Hence, given the high risk of failure and uncertainty of R&D investment (e.g., [15]), promotion-focused CEOs are more likely to pay attention to the potential positive outcomes of R&D investment, whereas prevention-focused CEOs are more likely to pay attention to the risk of failure associated with the resource allocation.

#### 2.2.2. Temporal Focus

How CEOs perceive the time horizon (i.e., temporal focus) has a significant influence on firm-level decisions [42]. A temporal focus can be defined as “the extent to which people pay attention to the past, present, and future” [43] (p. 4). Specifically, because R&D investment requires a willingness to invest with a long-term perspective [2], CEOs’ temporal dispositions play important roles, especially in setting objectives and providing motivation. A CEO’s perception of time serves as a filter that shapes expectation and assessment of strategic decisions and forms the basis of resource-allocation decisions [44]. Das [45] (p. 58) specifically wrote that “temporal dimension deserves comprehensive study because it constitutes a fundamental dimension of strategy making.”

Individuals have distinctive perspectives on time, namely, on the psychological past, present, and future [46]. Individuals with a strong focus on the past tend to make decisions based on repeated use and past memories [47], whereas individuals with a strong focus on the present set the current time frame (i.e., the here and now) as a key reference point in decision making [48]. In contrast, individuals with a strong future focus tend to envision future events [49], and thus are more likely to prepare to capture future opportunities. Given that the outcome of R&D investment is likely to be realized in the long term [2], it can be regarded as a future-oriented decision. As a result, future focus is likely to direct CEOs to pay attention to the outcome of R&D investment with an investment time horizon, because CEOs with a strong future focus will put more weight on long-term rather than short-term outcomes.

#### 2.2.3. Aspiration Level

How firms behave as a response to performance feedback has been one of the main research questions in the literature based on the behavioral theory of the firm [5]. According to this view, managers use a particular aspiration as a reference to assess their own firm’s performance [50,51]. Specifically, past performance often serves as the aspiration level when evaluating whether it satisfies the aspiration level, and based on this evaluation, managers engage in interpretations and make subsequent changes [52]. If there is any negative attainment discrepancy (i.e., firm performance below the aspiration level), managers shift the focus of attention, alter their level of effort, and search for solutions [53,54,55]. Previous studies found that performance feedback relative to aspiration level affects a variety of firm-level decisions such as strategic change [50], market entry [53,56], and acquisitions [29]. In particular, several studies [1,4,16,57] examined a firm’s response in the context of R&D investment.

The attention-based view [17,58] suggests that managers shift their focus of attention depending on the level of negative attainment discrepancy. Specifically, March and Shapira [19] proposed distinctive patterns for when managers deal with negative attainment discrepancy depending on the severity of discrepancy: (a) focusing on aspirations vs. (b) focusing on survival. When CEOs perceive a modest deficiency between aspirational and actual performance, they try to close the gap by conducting search behavior (i.e., problemistic search). In contrast, when decision makers focus on survival due to an excessive performance gap (i.e., financial distress), they are likely to reduce high-risk actions and avoid new strategic initiatives [29,59]. Instead, CEOs tend to preserve resources and tighten cost controls if they face a high level of negative attainment discrepancy. The threat from poor performance leads a firm to take a conservative approach [60]. To sum up, negative attainment discrepancy does not function as a “universal change trigger” [61]; rather, it encourages managers to focus on different objectives (i.e., aspiration vs. survival) depending on the degree or severity of the attainment discrepancy.

## 3. Hypotheses Development

### 3.1. Effects of Discretionary Slack on R&D Investment

R&D investment is often considered the main input in the innovation process [62]. It is an important investment decision for CEOs because overspending on R&D becomes a financial burden for a firm, whereas underspending on R&D leads to a loss of competitive advantage [1]. Because R&D investment is associated with high levels of outcome uncertainty and a risk of failure [15,63], a high level of managerial discretion (e.g., how much uncertainty and risk the CEO is willing to take) is required when making R&D investment decisions. While there is no clear consensus regarding the effect of organizational slack on innovation (e.g., [2,3,30]), this paper assumes a positive relationship between discretionary slack and R&D investment for the reasons described below.

Discretionary slack enables managers to engage in risk taking, innovation, and experimentation [5], with less strict performance monitoring of uncertain projects [4]. Excess resources and safety nets stemming from discretionary slack allow firms to pursue projects (e.g., R&D investment) that require risk taking and a long-term time horizon. Hence, since discretionary slack could provide the necessary freedom and flexibility a manager needs to make use of resources [21], we argue that it thus allows the manager to invest in high-risk projects such as R&D investment (i.e., slack search). Simply put, discretionary slack serves as a source of funding for innovation-seeking activities. O’Brien [64] (p. 420) specifically argued that discretionary slack “helps to provide insulation against cash flow volatility and ensures that investments in R&D are maintained even during bad times.” Conversely, when there is a low level of discretionary slack, R&D investment is more likely to be vulnerable to cutbacks [4], as investment in R&D does not guarantee generating positive financial outcomes. Taken together, we hypothesize as follows:

**Hypothesis 1.**  *Discretionary slack is positively associated with a firm’s R&D investment.*

### 3.2. The Moderating Role of a CEO’s Cognition

As described above, discretionary slack alone does not fully explain what determines the level of a firm’s R&D investment. Rather, the effects of slack on a firm’s R&D investments, especially when managers have a high level of discretion over slack, vary according to the decision makers’ cognitive mindsets. To this end, this study attempts to explain the effects of discretionary slack on R&D investment by taking the roles of key decision makers’ psychological traits into account, specifically as they relate to the R&D investment context, such as *promotion focus*, *future focus*, and *aspiration level*.

In the previous literature, R&D investment was characterized by (a) a high level of outcome uncertainty (i.e., big gain or big loss) with substantial failure risk [15], (b) a long-term payoff period [2], and (c) search behavior as a response to performance feedback [1,4]. Thus, how sensitive CEOs are to potential gains (i.e., promotion focus) plays a significant role due to the R&D investment’s outcome-uncertain nature. In addition, given that the benefits of R&D investment are likely to be realized in the long term, CEOs who are paying greater attention to future outcomes (i.e., future focus) are more sensitive to decisions regarding how much slack resources should be allocated to R&D investment. Lastly, R&D investment is a response to feedback from a firm’s performance relative to expectations, and a CEO’s perceived gap between its actual performance and expected performance (i.e., aspiration level) plays an important role in allocating resources to a firm’s R&D investment.

#### 3.2.1. CEO’s Promotion Focus

The principle of regulatory focus suggests that there are two self-regulatory modes that are associated with how an individual seeks pleasure or avoids pain—promotion and prevention focus [32]. In general, a promotion focus is concerned with positive results of achievement, growth, and gain, while a prevention focus is concerned with the negative outcomes regarding safety, stability, and protection [36].

Given the risk-involved attributes and gain-seeking purpose [36,39] of R&D investment, we argue that the CEO’s promotion focus is theoretically more relevant to decisions about spending on R&D. People with a strong promotion focus are more concerned about achievement, growth, and gain, and thus they prefer risk-taking actions out of a feeling of eagerness. A number of studies (e.g., [35,36,39]) have shown that the promotion focus is especially related to gain-seeking activities. For example, CEOs with a strong promotion focus are more likely to make acquisition decisions [12]. In particular, Kashmiri and colleagues [35] found that a CEO’s promotion focus leads to an increase in a firm’s R&D intensity.

Since CEOs who are predominantly promotion-focused prefer to achieve gains through risk-taking actions [33], they are more likely to be open to high-risk decisions such as R&D investment with discretionary slack. This is because CEOs with a strong promotion focus put more weight on benefits from R&D investment over the risk of failure. In contrast, CEOs with a prevention focus are reluctant to allocate slack resources to R&D investment, as they pay more attention to the potential risk of failure in recouping their investment decisions. Therefore, when a firm has increased discretionary slack resources, the promotion-focused CEO is likely to allocate greater resources to R&D investments. As such, we hypothesize that the positive relationship between discretionary slack and R&D investment will be strengthened when the CEO has a strong promotion focus.

**Hypothesis 2.**  *A CEO’s promotion focus moderates the positive relationship between discretionary slack and a firm’s R&D investment, such that the positive relationship is stronger when the CEO has a strong promotion focus.*

#### 3.2.2. CEO’s Future Focus

People are motivated by the expectations of the outcomes of their actions [65], including when they obtain the outcomes (i.e., time horizon). Innovation requires a firm to have a forward-thinking culture with a long-term commitment to its efforts, since it normally takes quite a long time to benefit financially from R&D investment. Thus, how CEOs perceive time is particularly important in future-oriented decisions, as it shapes the temporal direction of CEO cognition. As such, we argue that the extent to which the CEO has a future-oriented mindset matters in decisions regarding how much discretionary slack is allocated to R&D investment. Thus, we focus on the idea of future focus, a CEO’s cognition related to envisioning events that are yet to occur [66] and seizing future opportunities [44].

Given the long-time horizon for R&D investment, CEOs’ psychological trait regarding “time” is a key factor that directly involves strategic decisions about R&D investment. Drawing from a temporal focus perspective (e.g., [48,49]), we predict that the CEOs’ future focus would interact with discretionary slack resources for R&D investment decisions. Previous studies (e.g., [44,67]) suggested that a CEO’s future focus has a significant impact on strategic decision making. Specifically, for example, Yadav et al. [67] argue that if the CEO has a future focus, he or she is more attentive to a firm’s innovation outcomes.

As such, we argue that a CEO’s future focus moderates the positive relationship between discretionary slack and a firm’s R&D investment. Since investment in R&D takes a long time to pay off [2], CEOs with a strong future focus would pay more attention to what their firms will gain from investment in the future, rather than how much they should spend for the other projects that may provide short-term outcomes (e.g., sales promotion). Thus, we hypothesize that the positive relationship between discretionary slack and a firm’s R&D investment will be strengthened when the CEO has a strong future focus.

**Hypothesis 3.**  *A CEO’s future focus moderates the positive relationship between discretionary slack and a firm’s R&D investment, such that the positive relationship is stronger when the CEO has a strong future focus.*

#### 3.2.3. CEO’s Aspiration Level

According to the behavioral theory of the firm, a firm’s relative performance with respect to its managerial aspiration level affects the firm’s resource-allocation decisions [5,52]. Aspiration-level performance serves as a sort of reference which is based on comparisons with internal sources such as past performance or external sources such as competitors’ performance [53,68]. Thus, aspiration-level performance can be assumed to be the ultimate performance level that CEOs may wish to achieve. In this regard, we set the firm’s aspiration level of performance as a desired end state.

When CEOs perceive a gap between their actual performance and aspiration-level performance (i.e., attainment discrepancy) [51], they adjust the level of R&D investment, as previous studies (e.g., [1,58]) have reported that R&D investment is a response to performance feedback. Thus, we argue that the positive relationship between discretionary slack and a firm’s R&D investment depends on the CEO’s aspiration level. Specifically, we argue that, based on the attention-based theory [17], a manager’s perceptions change depending on the level of negative attachment discretion. Previous studies (e.g., [29,58,69]) suggested that CEOs switch their attention focus from aspiration to survival when they perceive low performance as a threat to the firm’s operations.

As such, when the firm performs below the aspiration level (e.g., lower than the industry average or its own prior performance records), but the gap is achievable (i.e., moderate negative attainment discrepancy), CEOs are more likely to seek various options (e.g., increasing R&D investment) to improve performance [19]. However, if the gap between a firm’s performance and its point of reference is too large (i.e., extreme negative attainment discrepancy), CEOs might seek strategic options that have an immediate impact on performance (e.g., layoffs or divestiture), because a firm’s survival might be threatened [29,59] and R&D investment may not be ideal to minimize threats in a short period of time. This is because managers are more likely to be risk-averse when they are focused on the firm’s survival [54].

When CEOs perceive an addressable level of a performance gap with some discretionary slack, they do not have to be concerned about the firm’s survival and thus are aspirational in seeking strategic options involving risk taking. In other words, the attention-based theory [17] suggests that CEOs can be more aspirational when the firm’s performance is close to the aspiration level (i.e., moderate negative attainment discrepancy). In contrast, when the firm’s performance is much lower than the reference points (i.e., extreme negative attainment discrepancy), CEOs may be hesitant to allocate slack resources for R&D spending, given the outcome uncertainty of R&D investment. Therefore, we hypothesize as follows:

**Hypothesis 4.**  *A performance below the aspiration level moderates the positive relationship between discretionary slack and a firm’s R&D investment, such that the positive relationship is stronger when the CEO perceives a moderate negative attainment discrepancy (i.e., the actual performance is close to the aspiration level).*

## 4. Method

### 4.1. Sample

Our sample includes publicly traded manufacturing firms in the United States (two-digit SIC codes 20 through 39) for the years 2006 to 2010. For the data collection, we used a number of archival databases such as Compustat, Execucomp, and GMI Ratings. For the CEOs’ letters to shareholders, we manually collected these from each firm’s official website. Because of full data availability from the various data sources, our final sample includes 654 firm-year observations from 175 firms.

### 4.2. Variables and Measurement

#### 4.2.1. Dependent Variable

To measure the level of a firm’s R&D investment, we used industry-adjusted R&D intensity [70]. Industry-adjusted R&D intensity is calculated by subtracting the industry average R&D intensity from the focal firm’s R&D intensity. We used the two-digit SIC code for each firm to define the focal firm’s industry.

#### 4.2.2. Independent Variable

In order to measure discretionary slack, we used cash reserves in a given year, following previous studies [21,71]. Cash is easily deployed by managers, who can allocate it with a great degree of freedom. Discretionary slack was measured as the natural logarithm of cash since the distribution of the variable is positively skewed.

#### 4.2.3. Moderating Variables

We measured CEO cognition, specifically related to R&D investment decisions, by capturing three psychological traits: *CEO promotion focus*, *CEO future focus*, and *negative attainment discrepancy*.

We conducted a content analysis of CEO’s shareholder letters to measure the CEO’s promotion focus and future focus (e.g., [12]). CEOs’ letters to shareholders include their perceptions of the firm’s current situation and future plans. In addition, these letters are considered as a CEO’s public message about the firm’s major priorities [44]. For these reasons, content analysis of CEOs’ shareholder letters has been widely used in the field of management studies [40,44,72,73,74,75]. In this study, we took a cognitive-linguistic perspective to measure the CEOs’ regulatory focus (i.e., promotion and prevention focus) and future focus. The cognitive-linguistic perspective argues that a writer’s psychological attention can be deduced from the type of words he or she uses in the text [76]. In fact, this approach has been widely used in previous studies to capture a CEO’s psychological characteristics [12,40,44,72].

First, in order to measure CEO promotion focus, we followed Gamache et al.’s [12] procedure for capturing a CEO’s regulatory focus. We used the same set of word lists to calculate the ratio of promotion-oriented and prevention-oriented words (see [12]). The list contains 27 promotion-oriented words (e.g., accomplish, gain, obtain, toward, etc.) and 25 prevention-oriented words (e.g., accuracy, defend, loss, vigilance, etc.), and we added variations of each word, such as alternative tenses [12]. To do so, we utilized the Linguistic Inquire and Word Count (LIWC) text analysis software [76]. This package provides the ratio of target words to total words written in each letter. The LIWC word list has been validated by more than 24,000 writers and speakers over the past 25 years [44]. The CEO’s prevention focus was included in the statistical analysis as a control variable.

Our second moderating variable is CEO future focus. We also measured CEOs’ future focus by taking a cognitive-linguistic approach [76]. We used the dictionaries produced by Pennebaker et al. [76], which include a set of 97 words that indicate future focus (e.g., will, may, foresight). Similar to CEO regulatory focus, we calculated CEO future focus using the LIWC software, as the number of target words divided by total number words written in each CEO’s letter to shareholders.

Third, in order to measure negative attainment discrepancy, we followed a multi-step procedure [71]. First, following previous studies, we used return on assets (ROA) for organizational financial performance [71]. Second, we took the industry average as the reference point for performance when firms performed below the industry average. However, for firms that outperformed this average, we set the prior performance as the reference point, while multiplying their prior performance by 1.05, which signifies a 5% increase from the previous year [71,77]. It should be noted that past research has used 5% as a proxy to signify that most firms make moderate improvements on their past performance [71,77]. Lastly, we calculated the gap between a firm’s actual performance and referenced performances. To capture firm performance below the aspirational level, we took the absolute value if the differences were negative (i.e., negative attainment discrepancy), and otherwise set variables as 0 (e.g., [78]).

#### 4.2.4. Control Variables

Our study includes a number of control variables that might affect a firm’s R&D investment, which include firm, industry, and CEO characteristics. First, we controlled for Firm age because it has potential effects on a firm’s experience [9,79]. Firm age is measured as the number of years a firm has been operating. Since larger firms may have more resources that are required for R&D spending, we included Firm size as a control variable [6]. Firm size is measured as the logarithm of total sales. Profitability and debt affect a firm’s spending on R&D [80], thus we controlled for Return on equity (ROE) and debt ratio. ROE is measured by net income divided by total equity, and debt ratio is calculated by a firm’s long-term debt divided by its total equity. We also controlled for a firm’s marketing resources by including Advertising intensity [29]. Following previous studies, we calculated Advertising intensity as advertising expenditure divided by total sales. As fluctuations in financial outcomes influence R&D expenditure [80], we also controlled for the effect of a firm’s specific conditions, which are operating income growth and operating income fluctuation. We followed the two-step approach from Keats and Hitt [81], using a firm’s operating income against time, to measure a firm’s income growth and its fluctuations. Since previous studies (e.g., [82]) found that board composition influences R&D investment, we controlled for the proportion of outside directors. The proportion of outside directors is calculated by dividing the number of outside directors by the total number of directors on the board.

Previous studies have presented CEO characteristics as important determinants of a firm’s R&D investment decisions [6,79,83]. Therefore, we controlled for various CEO characteristics such as CEO age, tenure, duality, gender, compensation, and CEO prevention focus. Younger CEOs are more likely to actively engage in R&D investment [6], and thus we included CEO age. In addition, a CEO’s tenure influences his or her cognitive complexity [84], and a long tenure enhances a firm’s relative R&D spending [79]. Therefore, we also controlled for CEO tenure. For CEO duality, we coded 1 if a CEO also holds a chairperson position, and 0 otherwise. We also controlled for CEO gender as a dichotomous variable. Many previous studies (e.g., [85,86]) show that CEO compensation is positively related to risk taking. Therefore, we included CEO compensation by calculating the logarithmically transformed total cash pay, which is the sum of salary and bonus [87]. We also included CEO prevention focus as a control variable. Prevention focus is another type of regulatory foci, which is related to vigilance and assuring safety in the decision-making process [32]. Crowe and Higgins [33] argue that both the promotion and prevention regulatory focuses can influence patterns of decision making. Similar to the CEO promotion focus, the CEO prevention focus is measured by conducting a content analysis of each firm’s CEO shareholder letters.

Lastly, the effects of industry and year are controlled by creating dummy variables. However, for the sake of brevity, we did not report the coefficients and standard errors of each industry and year dummy variables.

#### 4.2.5. Correction for Endogeneity Problem

Since it is possible that slack resources are affected by firm and industry characteristics, we addressed the possible endogeneity problem by using a two-step procedure [11,13]. First, we regressed discretionary slack resources against firm characteristics such as firm size, firm age, debt ratio, and industry characteristics such as industry munificence, dynamism, and concentration. The predicting model is significant (Wald Chi2 = 342.05, *p* ≤ 0.001) (see Appendix A). Second, following previous studies, we took only the statistically significant determinants and calculated the predicted value of discretionary slack resources. We included this variable as an endogeneity control variable in our main regression models.

### 4.3. Statistical Analysis

Our final dataset includes 654 firm-year observations from 175 firms. Since our dataset includes both time-series and cross-sectional components, ordinary least square (OLS) regression analysis is not appropriate. Therefore, we used the generalized estimating equations (GEE) model to test our hypotheses, following previous studies (e.g., [88,89]). The GEE model offers several advantages over random- or fixed-effect models, which are usually used in panel data analysis. First, it provides more robust coefficients when the dependent variable has a possibility of auto-correlation [90]. Such an advantage fits our study because our dependent variable might have a high propensity of auto-correlation. For example, firms’ R&D investment may show similar patterns by industry or time period. Second, GEE does not need to assume the normal distribution of the dependent variable [91]. As GEE estimation does not imply strict distribution assumptions, it is appropriate for this study, given that a firm’s R&D intensity data may not satisfy the assumption of normality. Lastly, GEE analysis has more robust results than random- or fixed-effect models because it offers a multi-matrix structure that fits the dataset appropriately [90]. Thus, we believe that GEE estimation would produce reasonable estimates of model parameters in this study. Further, we evaluate the multicollinearity problem by examining variance inflation factors (VIFs). The mean VIF value is 1.49 and it ranges from 1.03 to 3.12. The range of VIFs is far below 10, which is the conventional threshold [92], and thus we do not have multicollinearity issues.

## 5. Results

Table 1 shows the means, standard deviations, and correlations for the sample. Table 2 presents the results of the GEE analysis. Model 1 examines the effects of control variables on a firm’s industry-adjusted R&D intensity. Model 2 tests the main effect of discretionary slack on a firm’s R&D intensity. As predicted, discretionary slack has a positive effect on a firm’s R&D intensity (β = 0.18, *p* = 0.047), and thus Hypothesis 1 is supported. We also conducted additional tests to examine whether discretionary slack has a curvilinear relationship with R&D investments using its squared term. However, the results were not statistically significant.

**Figure 1:**
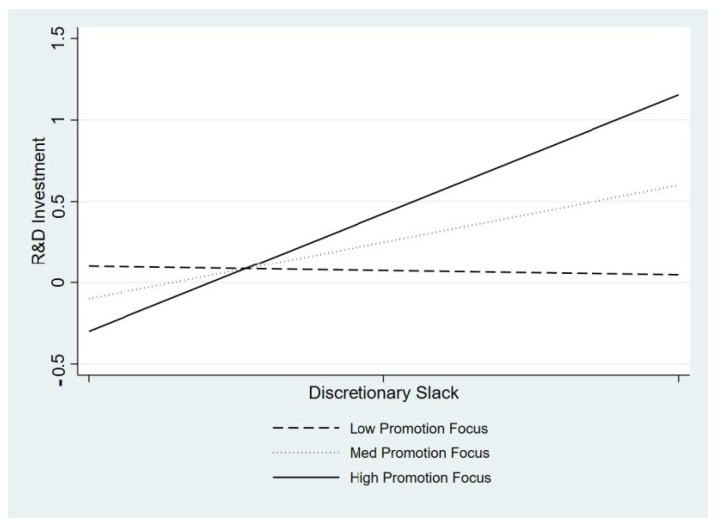
Effects of discretionary slack on R&D investment at different levels of CEO promotion focus.

To test the moderating effects, we include the interaction terms of discretionary slack and each component of the CEO cognition variables in Models 3, 4, and 5. All variables are mean-centered to test the interaction terms. First, we predict that the positive relationship between discretionary slack and R&D investment is strengthened when the CEO has a high level of promotion focus. Model 3 shows that there is a statistically significant and positive interaction between discretionary slack and CEO promotion focus (β = 0.19, *p* = 0.014). The plot of this interaction term is presented in Figure 1, showing that the positive relationship between discretionary slack and a firm’s R&D intensity is more pronounced by CEO promotion focus. Thus, Hypothesis 2 is supported. Second, Model 4 tests the moderating effect of CEO future focus. We predict that a CEO with a strong future focus strengthens the positive relationship between discretionary slack resources and a firm’s R&D intensity. However, we do not find any support for the moderation effect of CEO future focus (β = 0.11, n.s.). Thus, Hypothesis 3 is not supported.

**Figure 2:**
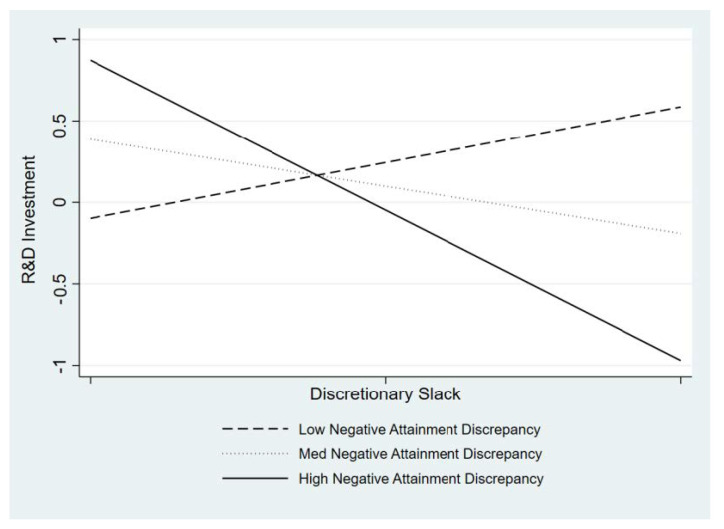
Effects of discretionary slack on R&D investment at different levels of negative attainment discrepancy.

Lastly, we hypothesize that the negative attainment discrepancy moderates the positive relationship between discretionary slack resources and a firm’s R&D intensity. As shown in Model 5, the interaction term is statistically significant (β = −1.59, *p* = 0.044). As shown Figure 2, when there is a moderate negative attainment discrepancy (i.e., the performance gap between aspiration and actual attainment is relatively small), there are positive effects of discretionary slack on R&D investment. However, the positive effect of discretionary slack on a firm’s R&D intensity is weakened as the level of negative attainment discrepancy (i.e., the firm’s performance is much lower than its reference point) increases. Rather, it shows the negative relationship (see solid line in Figure 2) when there is an extreme negative attainment discrepancy. This finding is consistent with the attention-based view argument indicating that managers shift their attention from aspiration to survival (i.e., reducing R&D investment). Thus, Hypothesis 4 is supported, as shown in Figure 2.

## 6. Discussion

This study examines whether the CEO’s psychological traits (i.e., promotion focus, future focus, and aspiration level), which are specifically relevant and important for CEOs in making R&D investment decisions, play moderating roles in making use of discretionary slack resources for innovation. While previous studies examined the roles of various types of CEO cognition, such as self-evaluation [93], narcissism [94], hubris [95], and extraversion [13] in a firm’s strategic decision making, in this paper, we selectively focused on cognitive traits directly related to R&D spending decisions. In previous studies (e.g., [2,4,6]), R&D investment has been characterized by (a) high outcome uncertainty, (b) a long-term payoff period, and (c) the organizational search process. Thus, we argue that how sensitive CEOs are to potential gain (i.e., promotion focus), how much they pay attention to future outcomes (i.e., future focus), and how much they perceive a gap between their firm’s actual and expected performance (i.e., aspiration level) matter in allocating discretionary resources to R&D investment, and we have some empirical support.

This study offers several important findings. For example, our findings show that CEO cognition plays a critical role in a firm’s decision making related to allocating discretionary slack resources. Overall, we find that a CEO’s promotion focus and aspiration level moderate the relationship between slack resources and R&D investment. Specifically, we found that the positive relationship between discretionary slack and R&D investment is more pronounced when a CEO has a strong promotion focus. Since a promotion focus is associated with gain-seeking and risk-taking actions, CEOs with a strong promotion focus may put more weight on achieving positive gains from spending on R&D and worry less about the potential risks (e.g., R&D failure).

We also find that the positive relationship between discretionary slack and a firm’s R&D investment is weakened when firm performance is far below that of its references (i.e., extreme negative attainment discrepancy). Previous studies (e.g., [55]) found that negative attainment discrepancy triggers an organizational search in response to performance feedback (i.e., problemistic search). However, our findings suggest that a firm’s behavior may vary depending on the types of search activities (e.g., R&D investment, acquisition, restructuring, etc.), as each action has distinctive characteristics, risks, and potential outcomes. Since CEOs set an aspirational performance level as a desired end state, they may wish to spend resources to close the gap. However, given the risks of R&D investment, CEOs would hesitate to spend limited resources on R&D investment when their firm performance lags far behind the aspiration level, because decision makers are likely to switch the focus of their attention from aspiration to survival (e.g., [19,29]). If a firm’s performance is below that of the reference points, particularly if performance is at a level at which the CEO believes it is a “feasible” solution to close the performance discrepancy, then the CEO would not be concerned about the firm’s survival per se, and thus would be aspirational in seeking riskier strategic options. If this is the case, risk-involved investments such as R&D investment could be an option they would consider. However, when the negative attainment discrepancy is too large, CEOs may cut R&D spending to deal with financial distress.

In addition, we assume that the CEO’s future focus would moderate the relationship between the discretionary slack resource and R&D spending. However, we did not find empirical support for this hypothesis. Why does a CEO’s future focus not play a significant role in this relationship? Indeed, this result may not be surprising, given that CEOs compare multiple long-term strategic options when they have sufficient discretionary slack resources. For example, slack resources can be invested in other long-term investment options, such as capital investment (e.g., plant modernization, capacity expansion, etc.), product or market diversification (e.g., acquisition, new market development), and formation of strategic alliances (e.g., joint ventures). Thus, although this study does not find empirical support, we do not believe that a CEO’s future focus is irrelevant in the relationship between discretionary slack and R&D investment. Rather, we call for future research that examines the roles of CEOs’ temporal focus, specifically future focus, in the effects of organizational slack on other long-term investment decisions, such as capital investment, diversification, and alliances.

### 6.1. Theoretical Contributions and Practical Implications

This study makes several theoretical contributions. First, it contributes to the growing body of literature on CEOs’ microfoundations by focusing on CEOs’ psychological traits related to the R&D investment decisions. Although many previous studies [6,79], based on upper echelons perspectives, have argued that CEO characteristics are important factors in organizational outcomes, recent studies (e.g., [96,97,98,99]) have increasingly paid attention to CEOs’ psychological and cognitive attributes. Our study empirically examines how a set of CEO cognitions—promotion focus, future focus, and aspiration level—interact with the discretionary slack resources to affect a firm’s R&D investment. In doing so, our study can offer more precise descriptions of how a CEO’s psychological traits interact with organizational slack, and how those interactions ultimately affect a firm’s input for innovation.

Relatedly, previous studies on organizational slack (e.g., [3,4]) are largely based on the behavioral theory of the firm [5] and focus on a firm’s process of performance evaluation, search behaviors, and decision making. Our findings complement the argument based on the behavioral theory of the firm by focusing on the roles of the psychological aspects of key decision makers. Although a number of studies (e.g., [2,4]), based on behavioral theory, examine the determinants of a firm’s R&D investment decisions, they often fail to take the roles of decision makers’ psychological aspects into account. Our findings suggest that inter-firm differences in R&D decisions may stem from the individual differences in CEOs’ psychological characteristics. In this regard, our study offers a more precise picture of the impact of slack resources on a firm’s decision-making process.

Third, our study shows that CEOs are likely to increase R&D investment when they perceive an addressable (rather than extreme) negative attainment discrepancy. Our empirical findings suggest that key decision makers shift their managerial attention from aspiration to survival (or vice versa) depending on the level of negative attainment discrepancy. The behavioral theory of the firm suggests firms alter their level of effort and search for solutions to fill the discrepancy (i.e., problemistic search). However, our findings show that CEOs try to conserve discretionary slack resources when they perceive a high level of negative attainment discrepancy. In this sense, we show how the microfoundations perspective, specifically, the attention-based view [17], may complement the argument based on the behavioral theory of the firm.

This study also points to some practical implications. We find that the effect of slack resources on a firm’s R&D investment can vary. Our results show that simply having slack is not a sufficient precursor for a firm increasing its R&D investment. What matters in promoting R&D investment is the extent to which the decision makers are (un)willing to allocate resources to spend on input for innovation. Some CEOs may immediately spend discretionary slack resources on R&D investment, whereas other CEOs may not, due to their psychological characteristics. R&D investment decisions are an outcome of discretionary slack interacting with CEOs’ psychological characteristics relevant to R&D. Specifically, our study shows that CEOs with a strong promotion focus and a reachable performance gap from the aspiration level are more likely to make use of resources for R&D. Thus, understanding CEOs’ psychological foundations offers a more precise prediction of how firms make strategic decisions.

### 6.2. Limitations and Future Studies

Although our findings make theoretical and practical contributions, this study is not without limitations. First, we measured the CEO’s promotion focus and future focus by analyzing CEOs’ letters to shareholders, following previous studies (e.g., [40,44,72,74]). Nonetheless, there might be a better way to measure an executive’s cognitive mindset. A follow-up study could examine the relationship between slack and R&D investment decisions using alternative methods for measuring CEOs’ psychological characteristics, such as analyzing CEOs’ speech and interviewing executives.

Second, the effects of different types of slack resources on a firm’s R&D investment may vary with different firm-specific contexts [57,100,101]. As such, future researchers may benefit from exploring potential situational and institutional factors, other than CEOs’ psychological traits, that could possibly moderate the relationship between discretionary slack and R&D spending. For example, the impact of slack resources on innovation may differ according to the industry competitiveness or regulatory environment.

Lastly, our sample consists of publicly traded manufacturing firms in the United States. As such, generalizing the findings of this study may call for caution, especially given that previous studies (e.g., [80]) have reported that the way firms make R&D investment decisions varies from country to country. Thus, the relationship between discretionary slack and a firm’s R&D investments might differ if, for example, samples are collected from other countries or different types of firms. Future studies need to examine the relationship between slack resources and R&D spending in different research contexts.

## 7. Conclusions

Resource allocation in R&D investment contributes to heterogeneity in a firm’s long-term competitiveness and financial performance. Thus, it is important to understand how R&D investment decisions are made. In this paper, we examine the effects of discretionary slack resources on a firm’s R&D investment by taking a set of CEO psychological traits into account. Our findings suggest that the level of discretionary slack and a CEO’s psychological traits (i.e., promotion focus and aspiration level) jointly affect a firm’s investment decisions on innovation input. From these results, our findings shed additional light on how CEOs’ psychological aspects play a role in these investment decisions. We make several contributions to the existing research on the behavioral theory of the firm, the microfoundations of strategy, and the attention-based view.

## Figures and Tables

**Table 1:** Descriptive statistics: Means, standard deviations, and correlations.

Variable	Mean	S.D.	1	2	3	4	5	6	7	8	9	10	11	12	13	14	15	16	17	18	19	20	21	22
1. R&D investment	0.00	5.49																						
2. Firm age	84.88	47.00	−0.24																					
3. Firm size	8.78	1.10	0.06	0.18																				
4. Return on equity	0.22	3.33	0.07	−0.02	−0.01																			
5. Debt ratio	−0.16	26.57	−0.01	0.00	−0.02	0.78																		
6. Advertising intensity	0.01	0.03	−0.03	0.16	0.16	0.05	−0.01																	
7. Industry concentration	0.33	0.15	0.00	−0.06	−0.16	0.00	0.01	−0.05																
8. Industry munificence	1.05	0.04	0.00	0.01	0.07	0.03	0.06	0.07	−0.26															
9. Industry dynamism	1.02	0.10	0.00	0.04	0.01	−0.01	0.00	−0.01	0.30	−0.51														
10. Operating income growth	1.14	0.42	0.12	−0.16	0.01	0.01	0.01	−0.08	0.00	0.23	−0.09													
11. Operating income fluctuation	1.06	0.17	0.05	−0.09	−0.09	−0.02	0.01	−0.05	0.10	−0.01	0.08	0.31												
12. Proportion of outside directors	0.87	0.06	−0.03	0.01	0.10	0.00	0.06	−0.14	−0.01	−0.07	0.08	−0.02	0.02											
13. CEO age	55.50	5.59	0.06	0.08	0.12	−0.01	−0.04	0.00	0.05	−0.07	0.11	0.01	0.05	0.08										
14. CEO tenure	6.10	4.17	0.04	−0.06	−0.02	−0.02	−0.01	−0.03	−0.02	0.00	−0.02	0.00	0.02	−0.01	0.37									
15. CEO duality	0.96	0.19	−0.17	0.14	0.09	−0.09	0.01	−0.15	0.05	−0.10	0.14	−0.02	0.03	0.06	0.14	0.04								
16. CEO gender	0.97	0.16	0.05	−0.05	−0.18	−0.01	−0.01	−0.03	0.05	0.06	−0.12	0.01	0.03	−0.02	0.11	0.02	−0.03							
17. CEO compensation	7.06	0.47	−0.02	0.07	0.60	−0.01	−0.03	0.16	−0.13	0.10	−0.06	−0.01	−0.06	0.05	0.13	0.02	0.07	−0.08						
18. CEO prevention focus	0.19	0.21	0.04	0.04	0.14	−0.04	−0.06	−0.12	0.01	0.02	0.01	0.02	0.01	0.06	0.09	0.01	0.07	0.02	0.06					
19. Endogeneity control	6.15	0.99	0.13	−0.06	0.94	−0.01	−0.03	0.10	−0.10	−0.16	0.21	0.01	−0.05	0.11	0.12	0.00	0.10	−0.19	0.55	0.13				
20. CEO promotion focus	1.19	0.48	−0.10	0.04	−0.14	−0.03	−0.03	−0.04	0.02	−0.06	0.02	−0.08	−0.06	0.02	0.01	0.07	−0.07	−0.04	−0.09	−0.03	−0.14			
21. CEO future focus	1.07	0.47	−0.03	0.02	0.03	−0.01	−0.03	−0.02	0.02	−0.01	−0.04	−0.02	−0.08	0.01	−0.07	0.01	−0.01	−0.02	0.05	−0.03	0.03	0.03		
22. Negative attainment discrepancy	0.02	0.05	0.13	−0.07	−0.05	0.03	0.02	−0.02	0.11	−0.04	0.02	0.02	−0.02	−0.02	0.01	0.01	0.05	0.02	0.00	0.00	−0.02	−0.06	−0.08	
23. Discretionary slack	6.11	1.46	0.33	−0.02	0.71	0.00	−0.01	0.10	−0.11	−0.06	0.07	0.07	−0.01	0.03	0.10	−0.01	0.02	−0.15	0.44	0.13	0.74	−0.15	0.02	0.08

Notes: Correlations greater than |0.05| are significant at *p* ≤ 0.05 and those greater than |0.07| are significant at *p* ≤ 0.01. Two-tailed coefficient test (n = 654).

**Table 2:** GEE Regression Analysis: Slack, CEO Psychological Traits, and R&D Investment.

Variables	Model 1		Model 2		Model 3		Model 4		Model 5	
	β	S.E.	β	S.E.	β	S.E.	β	S.E.	β	S.E.
Constant	−0.22	(3.27)	−0.05	(3.24)	−0.09	(3.24)	−0.10	(3.24)	−0.10	(3.24)
Firm age	−0.04 ***	(0.01)	−0.04 ***	(0.01)	−0.04 ***	(0.01)	−0.04 ***	(0.01)	−0.04 ***	(0.01)
Firm size	0.48	(0.99)	0.40	(1.02)	0.58	(1.01)	0.42	(1.02)	0.38	(1.01)
Return on equity	0.13 ***	(0.03)	0.13 ***	(0.03)	0.14 ***	(0.03)	0.14 ***	(0.03)	0.13 ***	(0.03)
Debt ratio	−0.01 ***	(0.00)	−0.02 ***	(0.00)	−0.02 ***	(0.00)	−0.02 ***	(0.00)	−0.02 ***	(0.00)
Advertising intensity	15.33 ^†^	(8.87)	13.13	(9.08)	13.07	(8.97)	13.39	(9.08)	13.69	(8.98)
Industry concentration	−0.94	(0.98)	−1.01	(1.02)	−1.24	(1.00)	−1.07	(1.02)	−1.01	(1.00)
Industry munificence	1.50	(6.27)	1.72	(6.49)	0.86	(6.38)	1.62	(6.49)	1.64	(6.38)
Industry dynamism	16.30	(17.37)	17.18	(17.98)	19.35	(17.67)	17.62	(18.00)	16.85	(17.69)
Operating income growth	0.46 ***	(0.14)	0.45 **	(0.14)	0.45 **	(0.14)	0.46 ***	(0.14)	0.46 ***	(0.14)
Operating income fluctuation	−1.11 **	(0.42)	−1.12 **	(0.43)	−1.15 **	(0.43)	−1.14 **	(0.43)	−1.24 **	(0.43)
Proportion of outside directors	−1.15	(1.29)	−0.97	(1.33)	−1.07	(1.31)	−1.11	(1.36)	−1.03	(1.31)
CEO age	−0.03	(0.02)	−0.03	(0.02)	−0.03	(0.02)	−0.03	(0.02)	−0.02	(0.02)
CEO tenure	0.02	(0.02)	0.02	(0.02)	0.03	(0.02)	0.02	(0.02)	0.02	(0.02)
CEO duality	−0.46	(0.37)	−0.51	(0.39)	−0.46	(0.38)	−0.49	(0.39)	−0.47	(0.38)
CEO gender	−0.07	(0.82)	−0.11	(0.84)	−0.05	(0.83)	−0.09	(0.84)	−0.15	(0.83)
CEO compensation	0.19	(0.17)	0.15	(0.18)	0.13	(0.17)	0.14	(0.18)	0.17	(0.17)
CEO prevention focus	−0.17	(0.32)	−0.16	(0.33)	−0.15	(0.32)	−0.16	(0.33)	−0.17	(0.32)
Endogeneity control	−0.06	(1.09)	−0.09	(1.12)	−0.24	(1.11)	−0.11	(1.13)	−0.09	(1.11)
CEO promotion focus	0.13	(0.12)	0.12	(0.12)	0.17	(0.12)	0.13	(0.12)	0.13	(0.12)
CEO future focus	−0.10	(0.12)	−0.08	(0.12)	−0.07	(0.12)	−0.08	(0.12)	−0.08	(0.12)
Negative attainment discrepancy	−1.17	(0.90)	−1.27	(0.93)	−1.35	(0.92)	−1.29	(0.94)	−0.73	(0.96)
Discretionary slack			0.18 *	(0.09)	0.17 *	(0.09)	0.18 *	(0.09)	0.17 ^†^	(0.09)
Discretionary slack × CEO promotion focus					0.19 *	(0.08)				
Discretionary slack × CEO future focus							0.11	(0.09)		
Discretionary slack × Negative attainment discrepancy									−1.59 *	(0.79)
Wald Chi-Square (d.f.)	99.86(39) ***		99.22(40) ***		107.84(41) ***		100.75(41) ***		105.66(41) ***	

Notes: ^†^ ≤ 0.10, * *p* ≤ 0.05, ** *p* ≤ 0.01, *** *p* ≤ 0.001. Two-tailed coefficient test; regression coefficients are unstandardized with standard errors in parentheses.

## Data Availability

Authors may provide data upon request.

## Appendix A. Correction for Endogeneity

**Table:**

	β	S.E.
Constant	6.15 ***	(0.07)
Firm size	0.90 ***	(0.06)
Firm age	−0.00 **	(0.00)
Industry munificence	−4.89 ***	(1.17)
Industry dynamism	12.03 **	(3.72)
Industry concentration	−0.26	(0.34)
Debt ratio	0.00	(0.00)
Wald Chi-Square (d.f.)	342.05(8) ***	
Number of observations	654	

Note: ** *p* ≤ 0.01, *** *p* ≤ 0.001.
